# A Hydrogel-Based Multiplex Coculture Platform for Retinal Component Cells

**DOI:** 10.1021/acsabm.4c01376

**Published:** 2025-01-16

**Authors:** Mohammad Haroon Qureshi, Ecem Metin, Cem Kesim, Ziba Zakeri, Baseerat Rumman, Afsun Sahin, Savas Tasoglu, Murat Hasanreisoglu, Emel Sokullu

**Affiliations:** † Koç University Translational Medicine Research Center, Koç University, Istanbul 34450, Turkey; ‡ Dept. of Molecular Biology and Genetics, Boğaziçi University, Istanbul 34342, Turkey; § Dept. of Ophthalmology, Koç University Hospital, Istanbul 34450, Turkey; ∥ Dept. of Mechanical Engineering, Koç University, Istanbul 34450, Turkey; ⊥ Dept. of Biophysics, Koç University School of Medicine, Istanbul 34450, Turkey

**Keywords:** hydrogel, multiplex platforms, retinal pigment epithelium, müller cells, coculture, spheroids, extracellular matrix

## Abstract

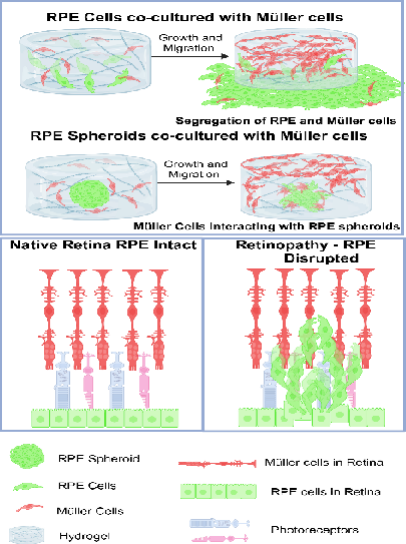

There is growing interest in generating in vitro models of tissues and tissue-related diseases to mimic normal tissue organization and pathogenesis for different purposes. The retina is a highly complex multicellular tissue where the organization of the cellular components relative to each other is critical for retinal function. Many retinopathies arise due to the disruption of this order. In this study, we aimed to generate a coculture model of retina-derived cells, namely RPE and Müller cells, in multiplexed 3D hydrogels. Using methacrylated gelatin (GelMA)-based 3D hydrogels, we compared the behavior of RPE and Müller cells when they were cultured together. These patterned multiplex hydrogels containing cells were cultured for several days to reflect how cells would reorganize themselves in the presence of another cellular component derived from the same tissue. Here, we present a multicellular multiplex platform for the creation of cellular networks with cells of retinal tissue that can be easily adapted to create more complex tissue-like alternatives for large-scale tissue modeling and screening purposes. We also present an alternative method of coculture by generating spheroids from one of the components while keeping the other component free and motile in the hydrogel. The latter model predicts enhanced possibilities of cellular interactions by retarding the movement of one of the component cells.

## Introduction

The retina is the inner photosensitive layer of the eye that is responsible for photoreception and the primary neural signal processing of the visual system. The retinal architecture features a complex and sophisticated cellular organization, which consists of a neural network for visual processing, glial and epithelial cells for retinal metabolism, and a double-sourced vascular system for blood supply.^1^

The retinal neural circuitry is composed of a three-order organization called the neurosensory retina: (a) photoreceptor cells, (b) bipolar cells, and (c) retinal ganglion cells (RGCs). The retinal metabolism and integrity are mainly regulated by retinal pigment epithelial (RPE) cells and Müller glia. RPE cells provide a metabolic hub for the photoreceptor cells on the outer border of the neurosensory retina and are responsible for a multitude of functions related to photoreceptor metabolism, including outer segment renewal, visual pigment regeneration, and barrier function,^2^ whereas Müller cells are the major glial cells for the structural scaffolding of the retina by extending through all layers of the neurosensory retina. For their major supportive functions, Müller cells are in close contact with the retinal neurons as well as the blood vessels. Müller cells support the normal function of retinal neurons through mediating metabolic homeostasis. The retinal pigment epithelium is the single-cell-thick layer of polygonal cells that constitute the outermost layer of the retina. While the inner side of RPE cells is connected to and supports the outer segment of the photoreceptors, the outer layer interacts with the choroid and Bruch’s membrane. Constituents of the retina and their proper organization within the tissue are paramount for its function, and any disruption may lead to retinopathies. Given the seminal role that the Müller cells and RPE cells play in the core structural integrity and functional harmony of the retina, the majority of retinopathies and associated pathophysiological mechanisms are mainly related to primary functional and structural disturbances of the retinal support system provided by Müller cells, RPE cells, and the inner and outer blood–retinal barriers. The overload of oxidative stress-induced damage results in the depletion of pericytes and disruption of the inner blood–retinal barrier in retinal vascular diseases, including diabetes and retinal vascular occlusions. The metabolic overload of RPE, along with the oxidative disruption of the RPE-Bruch membrane complex and the outer blood–retinal barrier systems, leads to the accumulation of waste products and cellular debris, depletion of photoreceptor cells, and subsequent formation of immature new vessel formations (neovascularization) in the outer retina, a clinical spectrum of diseases that are coined with the definition of age-related macular degeneration.^3−5^

Proliferative vitreoretinopathies are characterized by the disruptive migration and proliferation of some components of the retina, including RPE and glial cells, with parallel accumulation of ECM proteins as retinal and vitreous membranes.^6^ Retinal layers get disrupted by glial cell proliferation, causing damage to the photoreceptors.^7^ Müller cells show signs of reactivity following retinal injury through upregulation of intermediate filaments Nestin, GFAP, Synemin, and Vimentin, suggesting the acquisition of a proliferative and developmentally immature state. Monitoring the interactions of this multilayered structure in the retinal plane in the culture environment may provide an important infrastructure platform for the study of the effects of retinal diseases and the proposed solutions at the cellular and molecular levels. In order to monitor these interactions in the in vitro environment by fixing certain parameters, the preferred 3D cell culture systems have come to the forefront in recent years. These 3D cell culture platforms are frequently preferred to elucidate cell proliferation, differentiation, and identification of growth factors, as well as to understand the relevant downstream pathways involved in the physiology of specific cell types under different pathological and physiological conditions and to determine the effects of drug candidate molecules on the cell–cell and cell–matrix interactions in disease models.^8^ Again, the coculture of two and/or more different cell types in 3D cell culture has gained importance in terms of providing a realistic representation of cell interaction effects and barrier models on tissue formation by mimicking the natural environment in the laboratory.^9^

Natural tissue compartments can be mimicked through 3D coculture protocols to observe the changing behavior of different cell populations with environmental factors and the effects of the extracellular environment on synthetic biology and tissue regeneration parameters.^10^ In the current study, we showed a multiplex hydrogel-based 3D coculture system of two components of the human retina, namely, the RPE and Müller cells. Using a multiplex GelMA-based hydrogel matrix, we show the biocompatibility of GelMA for these retinal components and their successful coculture. We observed a differential rate of cell migration out of the hydrogel onto the 2D culture surface, resulting in unequal cell retention in the hydrogel. The outward migration of the two cells was reduced with the use of a mixture of PEG and GelMA to make a precoating onto the cell culture surface before laying hydrogel patterns containing cells on top of them. The protein-repellent nature of PEG in the bottom layer reduced the cells’ escape from the hydrogels. RPE1 cells exhibited low retention in the 3D hydrogel and migrated out of the hydrogel. Müller cells also exhibited outward migration from hydrogels into the 2D surface but showed better retention within the hydrogel. In retinopathies, RPE cells exhibit migration away from their single-cell layered organization in the retinal epithelium and may show noncanonical organizations and interactions with other components of the retina. To exhibit the potential of our coculture system in disease modeling, we needed to circumvent the differential retention of cells by incorporating RPE1 cells in spheroids and coculturing them with freely migrating incorporated Müller cells in the hydrogel. RPE1 cells’ retention within spheroids delayed their motility and provided a prolonged period of possible hetero cell–cell interactions. We show that while the majority of the Müller cell population organized themselves along the boundary of the hydrogel, RPE1 cells exuding outward from the spheroids retained the Müller cells in their vicinity with interaction between the cells. Our system is thus a minimal model system of coculturing retinal component cells, with the possibility of generating more complex and multicellular assemblies for future applications.

## Methods

### Materials and Characterization

#### Hydrogel Form of GelMA Synthesis

Gelatin methacrylate (GelMA) is synthesized by modifying the One Pot Method, as previously described.^11^ Briefly, gelatin (10%) (w/v) in carbonate-bicarbonate buffer (0.25 M, pH = 9) is dissolved and stirred on a magnetic stirrer for 2 h at 45 °C. Methacrylic anhydride (MAA/gelatin = 0.1/1 mL/g)) is slowly added while the solution is stirred at 50 °C, where the reaction proceeds for 3 h. Then, the pH of the solution is adjusted to 7.4 in order to terminate the reaction. After being filtered, the solution is dialyzed using 12–14 kDa cutoff dialysis tubing for a week in distilled water to remove salts and unreacted compounds. The polymer solution is then lyophilized, and the resultant product is stored at −20 °C until further use.

#### Surface Treatment of Coverslips

The adhesion of GelMA to coverslips was enhanced by acrylation, which was performed using 3-(trimethoxysilyl)propyl methacrylate (TMSPMA) (Sigma-Aldrich, Cat. No. 440159). Coverslips were dipped in a 10% (w/v) sodium hydroxide (Merck, Germany) solution for 1.5 h, followed by washing with distilled water and drying in a vacuum oven. Then, the coverslips were treated with TMSPMA overnight at 80 °C. The treated coverslips were washed with absolute ethanol 3 times and allowed to dry within a laminar flow hood. The coverslips were kept at room temperature prior to the experiments and used within 1 week after acrylation.

#### Preparation of Prepolymer Solution

The prepolymer solution is prepared by using a photoinitiator (PI) 2-hydroxy-4′-(2-hydroxyethoxy)-2-methylpropiophenone (Irgacure 2959). The PI is dissolved in DPBS (1%) (w/v) at 80 °C. 5% (w/v) GelMA is added to the solution and incubated at 37 °C. The mixture is then filtered (0.2 μm) and either immediately used or stored at 4 °C.

#### Fourier-Transform Infrared Spectroscopy (FTIR)

The characterization of lyophilized GelMA is performed by obtaining the spectra of molecular absorption and transmission peaks from vibration frequencies between atomic bounds. Fourier transform infrared spectroscopy with attenuated total internal reflection (FTIR-ATR) was utilized to evaluate the chemical constituents of pure gelatin and methacrylated gelatin (GelMA). A Nicolet iS10 Thermo Scientific spectrometer with a diamond crystal at a nominal angle of incidence of 45° and a ZnSe lens was used. The spectra were recorded with 32 scans at a resolution of 4 cm^–1^ from 600 to 4000 cm^–1^.

#### Scanning Electron Microscopy (SEM)

The morphological properties of photo-cross-linked GelMA were observed using a Zeiss Ultra Plus Field-Emission Scanning Electron Microscope (FE-SEM). The photo-cross-linked GelMA was lyophilized for 24 h before imaging. Samples were sputtered with 10 nm of gold. Imaging was performed at an electron high tension (EHT) of 3.00 kV with a working distance (WD) of 5.7 mm. Quantification of pore sizes was performed using the image processing software ImageJ.

#### NMR Spectrometry

To assess the extent of functionalization of GelMA, proton nuclear magnetic resonance (^1^H NMR) spectra of gelatin and GelMA were recorded using a Bruker Avance Neo 500 MHz NMR spectrometer. The spectra were measured under ambient conditions. D_2_O was employed as the solvent, and the proton signal from remaining D_2_O served as a reference.

#### Cell Culture

RPE1 cells (ATCC:hTERT-RPE1-CRL-4000) were cultured using DMEM/F12 medium with 10% FBS and 1% penicillin–streptomycin. A self-immortalized Müller cell line (MIO-M1)^12^ was cultured using DMEM with 10% FBS and 1% penicillin–streptomycin. Cells were regularly checked for mycoplasma contamination.

#### Encapsulation of Müller and RPE1 Cells for MTT Assays

Müller and RPE cells are trypsinized when they reach 80% confluency and mixed within the prepolymer solution with a desired density of cells (4 million/mL). The GelMA suspension-containing cells on the curated coverslips are cured for 45 s using the OmniCure S2000 UV 12 Light Curing System (Excelitas Technologies Corp., Waltham, MA, USA) and doughnut-shaped photomasks from a distance of 50 mm, in which the power density was set to 5.25 W/cm^2^. After UV exposure, the non-cross-linked solution is washed away with DPBS. The desired amount of medium is added to encapsulated cells and put into an incubator until further assays.

#### MTT Assay

The viability of three-dimensional cell constructs was assessed at days 0, 1, 4, and 7 using MTT ((3-(4,5-dimethylthiazol-2-yl)-2,5-diphenyltetrazolium bromide) (Invitrogen, Cat. No. M6494). MTT stock solution (5 mg/mL) in DPBS was prepared and added to the serum-free medium at a concentration of 10% (based on the final volume of each well of a 6-well plate, which was 2 mL), and incubated at 37 °C for 4 h. The solution was removed, and 1 mL of DMSO (dimethyl sulfoxide) (PanReac AppliChem, Cat. No. A3672) was added. Incubation took place for 25 min prior to the measurement of optical densities at 570 nm, using the BioTek Synergy H1 Hybrid Reader.

#### Fluorescent Labeling of the Cells

HEK-293T cells were used to package empty EGFP (Addgene no. 17448) and RFP (Addgene no. 109377) plasmids into lentiviruses using psPAX2 and pMD2.G plasmids. Supernatants containing viruses were used for the transduction of RPE1 and Müller cells.

#### PEG/GelMA Coating of the Culture Surfaces

A solution of 50% PEG and 5% GelMA was mixed equally and plated on the glass bottom plate. This mixture was exposed to UV for 90 s. Subsequently, another layer of 5% GelMA was spread on top of the polymerized PEG/GelMA and exposed to UV for 150 s. This combination of layering was left under UV for 2 h and overnight, respectively, until it completely dried.

#### Multiplexed GelMA Encapsulation

**Figure 1:**
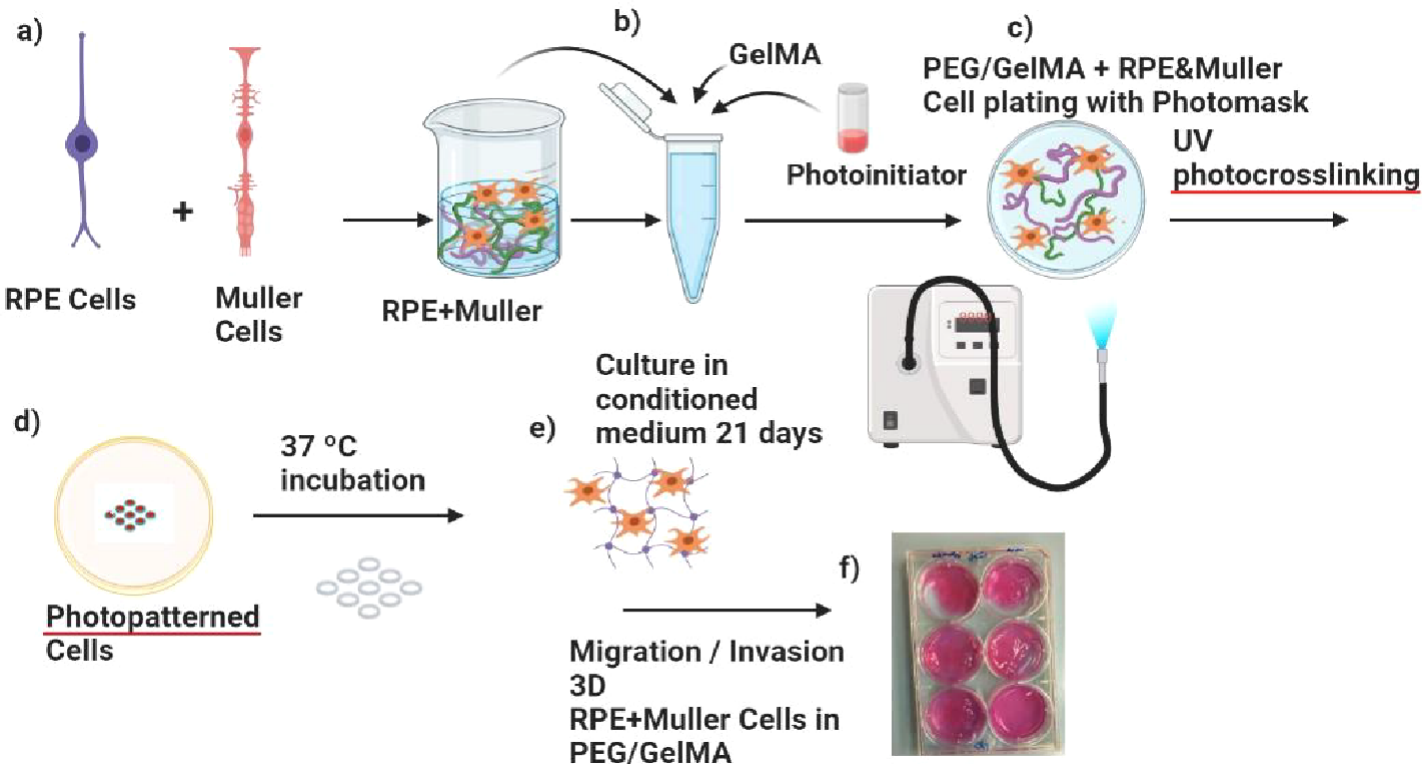
A schematic representation of the coculture of RPE and Müller cells in GelMA-based 3D hydrogels. (a) Cartoon representations of RPE and Müller cells, cultured and mixed in equal proportions. (b) GelMA with photoinitiator enables cross-linking. (C) GelMA mixed with RPE and Müller cells, when exposed to UV light, can polymerize the reactive GelMA trapping the cells inside the 3D hydrogel. A modification of the process involves adding an additional layer of PEG and GelMA on the surface, prior to the polymerization of the hydrogel. (d) Photomask-mediated UV exposure enables patterning of hydrogels in multiplex in-sample replicates. (e) Long-term coculture of the cell mix allows cellular autonomy to reorganize themselves in the hydrogel as the coculture matures. (f) Ability to observe cellular migration, reorganization, and morphological changes within the hydrogel over time.

The experimental scheme for encapsulation is shown in Figure 1. RPE1 cells expressing EGFP and Müller cells expressing RFP were mixed in equal proportions and resuspended in 5% GelMA with a photoinitiator at a density of 4 million cells/ml (Figure 1A,B). This GelMA-cellular mix was placed on culture surfaces coated with PEG/Gelma or left uncoated and exposed to UV light through photomasks to generate polymerized GelMA patterns of the respective shapes (Figure 1C,D). Polymerized GelMA patterns with the trapped cells inside were cultured for several days. A photomask was placed underneath the bottom of the dish. The suspension was exposed to UV light through a doughnut-shaped photomask from a distance of 50 mm, with the power density set to 5.25 W/cm^2^. Unpolymerized GelMA was washed off using DPBS and gentle agitation. Cells were observed in encapsulations, and culture medium was added (DMEM/F12).

#### RPE1 Spheroids and Coculture with Müller Cells

RPE1 cells expressing EGFP were counted and used in the hanging drop method. Spheroid formation was nearly uniform after approximately 10 days. Spheroids were gently collected and resuspended into GelMA containing single-cell Müller cells expressing RFP. Using hexagonal-shaped photomasks and a 35 mm distance, GelMA was polymerized onto glass-bottom confocal culture plates. Unpolymerized GelMA was washed off using DPBS and gentle agitation. DMEM/F12 culture medium was added to the culture plates and incubated at 37 °C.

#### Confocal Microscopy

A Leica TCS SP8 laser scanning confocal microscope was used for imaging of the encapsulated cells. GelMA-encapsulated cells were incubated at 37 °C and 5% CO_2_ during imaging. The tile scan function was used for imaging a large section of the multiplexed hydrogels with encapsulated cells. Z-stacks were used to create a 3D rendering of the sections. The LasX software was used for data processing after imaging.

#### Statistical Analysis

The whole absorbance results were processed via GraphPad Prism 8 software. Two-way analysis of variance (ANOVA) followed by a post hoc Tukey test was considered for statistical analysis of the cytotoxic test results. The level of significance was accepted to be *p* < 0.05.

## Results and Discussion

### FTIR and SEM Characterization of GelMA

**Figure 2:**
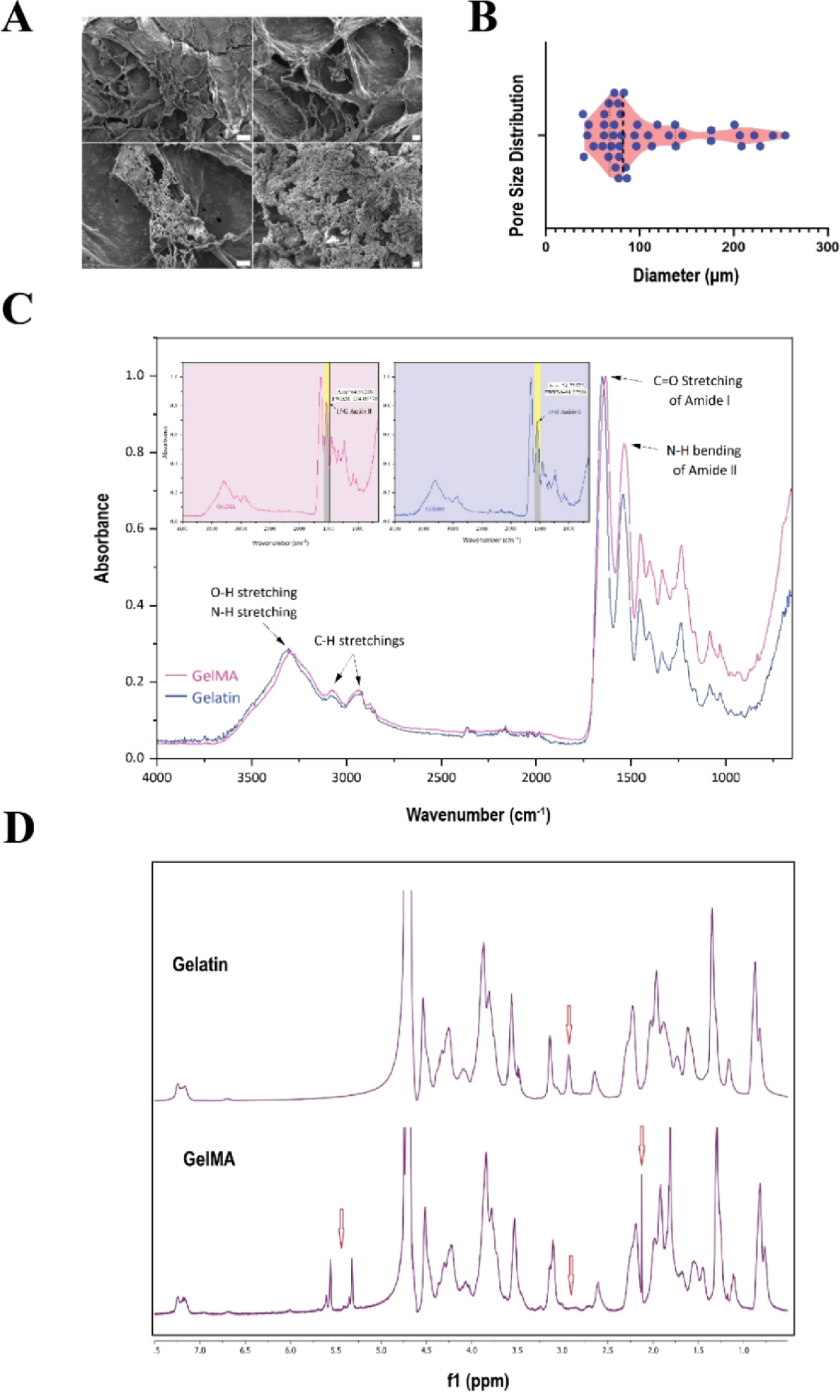
Analysis of GelMA. (A) SEM images of polymerized GelMA at different magnifications. Magnifications from top left, clockwise: 200×, 500×, 1000×, and 5000×. Scale bars from top left, clockwise: 100 μm, 20 μm, 20 μm, and 2 μm. (B) Distribution of pore size after the polymerization of GelMA, *n* = 44. (C) FTIR analysis of gelatin and GelMA. (D) Comparison of ^1^H NMR spectra of methacrylated gelatin (GelMA) with pure gelatin shows the appearance of the methacrylamide group (5.3–5.6 ppm) and the methyl protons of the methacrylate groups (∼2 ppm), as well as a decrease in the intensity of the peak associated with the ε-protons of lysine (∼3.0 ppm).

The morphology of the hydrogels was studied by scanning electron microscopy (SEM). From the images in Figure 2A, hydrogels display a porous structure showing continuity between pores, shown here at different magnifications. These features support the growth of cells in the scaffold owing to the appropriate size of the pores, reduced shear stress caused by fluid passage, and increased oxygen and nutrient transfer to the cells. The average pore size of the hydrogel under our experimental conditions is 108.67 μm (Figure 2B). In the FTIR spectrum, the peaks observed at 3080 cm^–1^ and 2945 cm^–1^ correspond to the stretching vibrations of C–H groups in alkenes and alkanes, respectively, indicating the presence of functional groups derived from gelatin and methacrylic anhydride. Additionally, the peak associated with amide II (∼1550 cm^–1^) is due to the bending vibrations of the N–H bond and stretching vibrations of the C–N bond in amides. The increase in intensity and area under the amide II peak in gelatin methacrylate (GelMA) compared to gelatin indicates a successful methacrylation process. This increase arises from the formation of new groups, such as methacrylate esters and modified amide bonds, through the reaction of primary amine (NH_2_) and hydroxyl groups in gelatin with methacrylic anhydride. These structural changes are clearly reflected in the intensity and position of the FTIR peaks.

### NMR Spectrometry

To validate the accurate synthesis of methacrylate gelatin and determine the degree of substitution (DS) of gelatin, ^1^H NMR spectrometry was employed. Figure 2D illustrates the ^1^H NMR spectrum of methacryloyl groups in comparison to that of pure gelatin. Given that lysine primary amine connected to gelatin serves as the principal site for gelatin substitution, the reduction in the integrated signal at approximately 3.0 ppm, corresponding to the methylene group of lysine (2H) in gelatin, was utilized for the purpose of this calculation. The signal at approximately 5.3–5.6 ppm corresponds to the acrylic protons (2H), while the signal around 2 ppm belongs to the methyl group (3H) of the bonded methacryloyl group.^13^ As an internal reference, the spectra were normalized using the aromatic moieties (5H) of phenylalanine signals, around 7.3 ppm, as they were not modified by MA during the reaction. Upon analysis of the related spectrum, it was determined that the DS of the produced GelMA is 70%.

### Biocompatibility of GelMA in the Retinal Component Context

**Figure 3:**
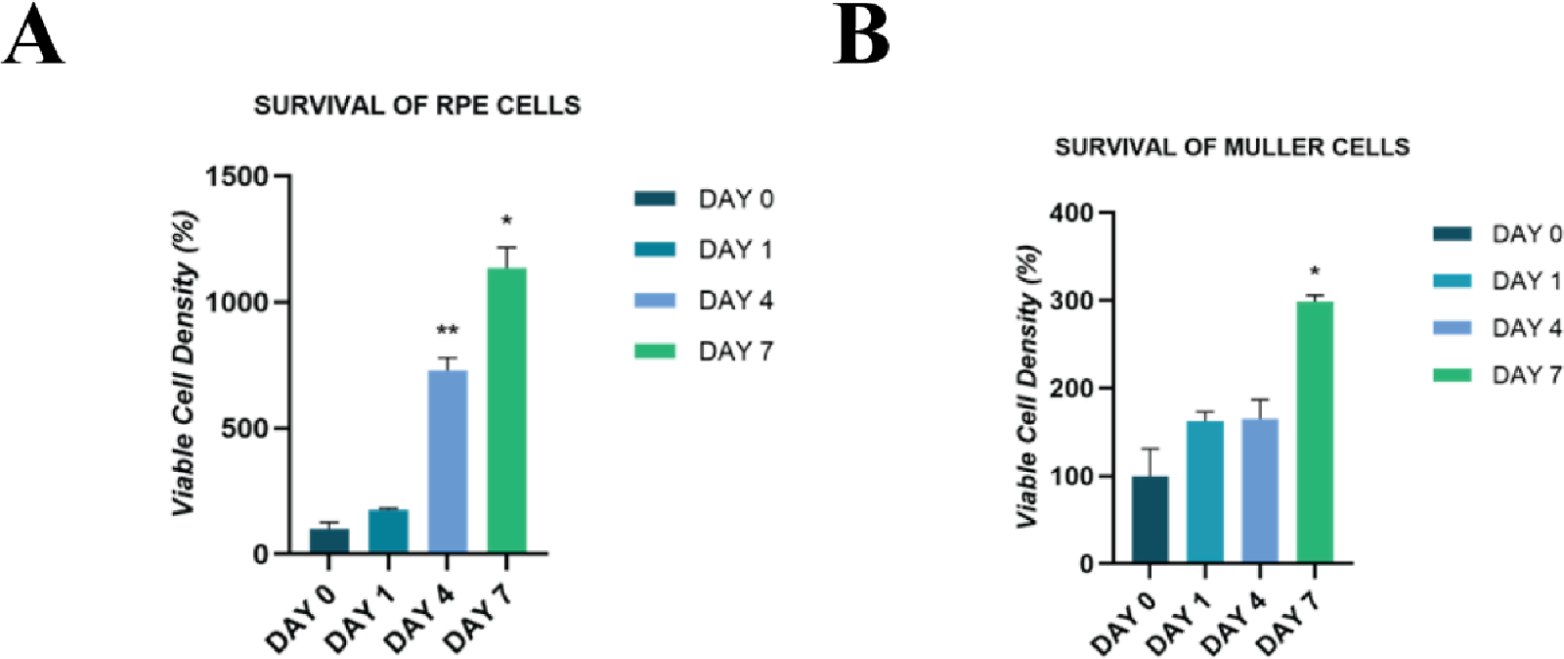
The viability assay of RPE1 and Müller cells in GelMA using the MTT assay. (A) Graphical representation of the survival trend of RPE1 cells growing in GelMA from the day of encapsulation (Day 0) to 7 days after encapsulation. (B) Graphical representation of the survival trend of Müller cells from the day of encapsulation (Day 0) to Day 7 after encapsulation. Initial density of cells = 4 million/ml of hydrogel. *n* = 3.

To perform a coculture of retinal component cells, we first decided to check the effects of the environment containing GelMA on the growth of RPE and Müller cells. We used RPE1, the immortalized human RPE1 cell line, and a self-immortalized Müller cell, MIO-M1 (hereinforth termed RPE and Müller cells). RPE and Müller cells were separately encapsulated into GelMA, and their progress was followed by a cell viability assay, MTT. Figure 3 shows the growth dynamics of RPE1 and Müller cells within 5% GelMA for a 7-day period. Despite the viable cell density (%) at Day 1 being similar in Müller and RPE cells (mean: 162.36 and 177.32, respectively), RPE cells established a nearly 4-fold increase in proliferation on Day 4 compared to that on Day 1 (*p* = 0.02). The difference in viable Müller cell density between Day 1 and Day 4 was deemed insignificant (*p* = 0.99, mean: 162.36 and 165.12, respectively). On Day 7, the fold change of cell viability compared to Day 4 appeared to be greater in Müller cells than in RPE cells (*p* = 0.37, fold change: 1.8 and 1.6, respectively). Both Müller and RPE cells exhibited a significant upward trend in cell survival over 7 days (*p* = 0.01 and 0.02, respectively). Although both cells are derived from the retina, they display different adaptation patterns to the hydrogel environment containing GelMA. The viability assay was performed on a minimum of 3 unique experiments from each cell line.

### Cellular Profiles in Coculture

There are noticeable differences between regular 2D monolayer cultures and 3D hydrogel-based cultures in terms of cell shape, density, as well as sensitivity to certain drugs.^14^ Tumor spheroids, e.g., 3D spheroids, better mimic solid tumors in terms of gene expression, spatial organization within the tumor, and drug resistance mechanisms, among others. In comparison with 2D cell culture models, 3D spheroids can accurately mimic some features of solid tumors, such as their spatial architecture, physiological responses, secretion of soluble mediators, gene expression patterns, and drug resistance mechanisms. These unique characteristics highlight the potential of 3D cellular aggregates to be used as in vitro models for screening new anticancer therapeutics, at both a small and large scale. Natural cell shape, growth patterns, and multilayered organization of cells are reflected better in 3D than in 2D.^15,16^ Unequal distribution of nutrients due to uneven exposure to the culture medium leads to an inactive cell core representing the tumor core.^15,16^ Cell–cell junctions are more common in 3D cultures, enabling communication through ions, small molecules, as well as electrical conductions.^15−19^ Cells exhibit better differentiation in 3D compared to 2D.^15,16,20^ In 3D cultures, cells exhibit more drug resistance, which reflects the natural behavior of tumors compared to 2D.^16,20^ The expression profiles of genes and proteins often better reflect the in vivo levels.^15,16,18^

We intended to perform cocultures of RPE and Müller cells in GelMA. To differentiate the two types of cells, we imparted two different fluorescent colors to them. Using lentiviral-mediated transduction, RPE were given EGFP and Müller cells were given RFP. These two different cell populations were mixed and pelleted in equal proportions and resuspended in liquefied GelMA. The cells were encapsulated in the hydrogel by exposing the mixture to UV light through a patterned, doughnut-shaped photomask. We observed significant migration of cells out of the hydrogel patterns on the cell culture surfaces. Cell culture-treated surfaces between the hydrogel patterns provide optimal attachment of cells to the 2D surface. Since the 2D surface allows higher migration and proliferation, cells start to cover the space surrounding the hydrogel patterns. We employed, in parallel, a separate encapsulation by first making a GelMA and PEG (PEG-8000) mix as a layer exposed to UV light for polymerization. Upon this relatively tougher bottom layer, the patterned hydrogel encapsulation was laid by using photomasks. The reason for using additional PEG/GelMA layers below the GelMA hydrogels was to discourage premature migration of cells out of the hydrogels and onto the 2D surface. PEG mediates the creation of cell-repellent surfaces on the culture dishes. PEG is an uncharged and hydrophilic material with low toxicity and low immunogenicity and is a preferred polymer for the prevention of protein adsorption. A mix of PEG/GelMA thus provides a less-than-optimal opportunity for cell attachment and migration. As the hydrogel/cellular assemblies age, cells tend to make their way out of the 3D hydrogel environment to a 2D surface of culture plates, where they find it more feasible to attach to the surface. A PEG/GelMA layer does not provide a layer as favorable for cells to attach as the regular cell culture-treated surface and would thus prevent cellular attachment to the cell culture plate. We followed the cellular behavior at 3 separate time points over 12 days post encapsulation using confocal laser scanning imaging with Z-stack.

**Figure 4:**
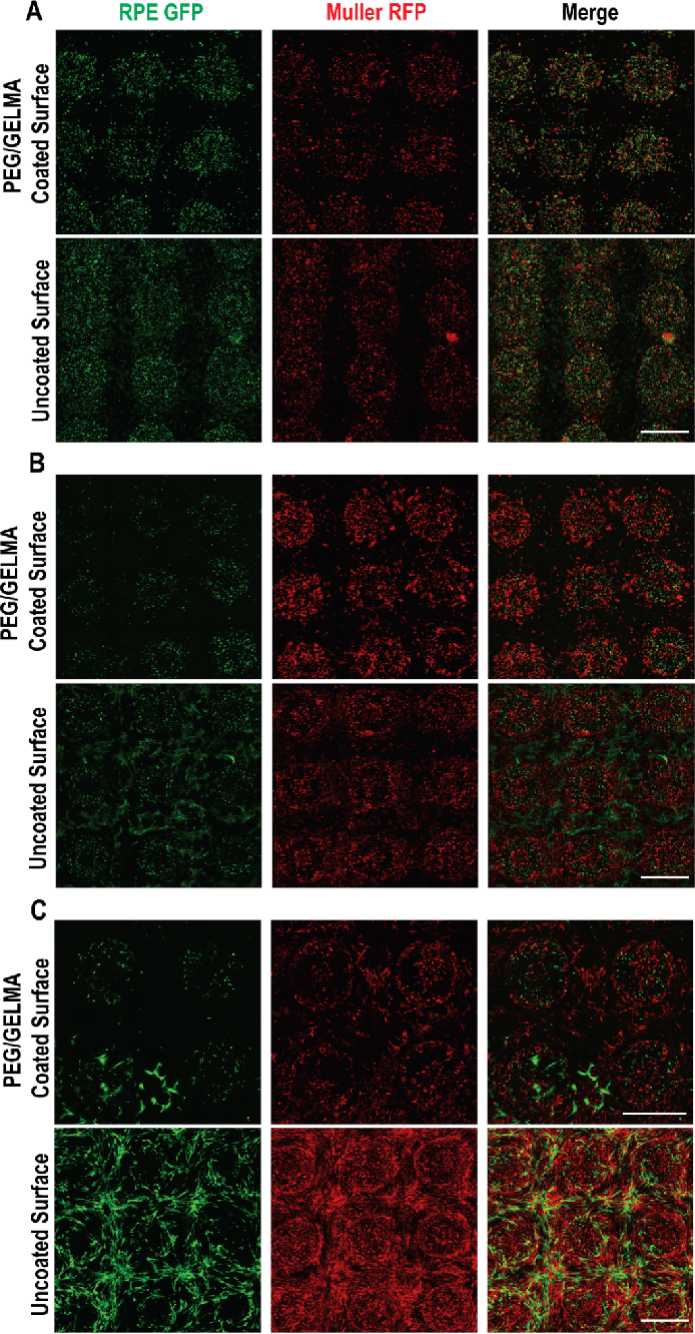
Relative organization of cells within the hydrogels. RPE1 cells (green) and Müller cells (Red). (A) Day 1, (B) Day 3, and (C) Day 12. The upper panels show PEG/GELMA-coated surfaces, while the lower panels show uncoated surfaces used from the hydrogel attachment alone. Scale bars: 1 mm.

**Figure 5:**
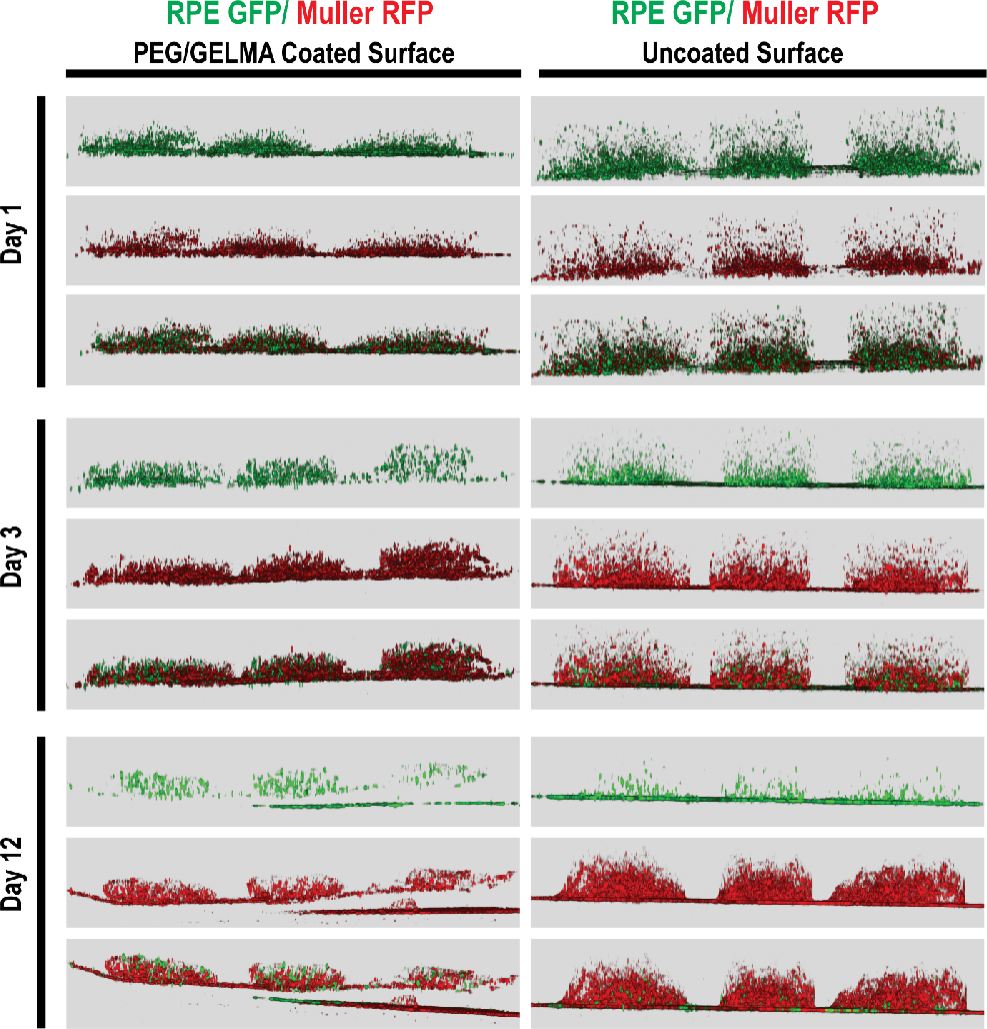
Relative 3D organization of cells within the hydrogels. RPE1 cells (green) and Müller cells (Red) on Day 1 (upper panels), Day 3 (middle panel), and Day 12 (lower panels) of coculture. The right panel shows the cellular presence upon the PEG/GelMA-coated surface, while the left panel shows uncoated surfaces.

Initially after the encapsulation, cells appeared to be small and rounded on Day 1 (Figure 4A). This essentially represents the the initial cellular profiles of encapsulation, where due to the lack of secreted extracellular matrix proteins native to the specific tissue and deficient integrin adhesions, cells are trapped in the hydrogel. Cells show minimal motility and proliferation at this stage. Both PEG/GelMA and the uncoated surface as a base appear to be similar in their appearance. Cells need secreted extracellular matrix as well as the formation of integrin attachments between cells and the hydrogel/ECM. ECM is secreted by the cells themselves in an innate tissue or in a cell culture setting. In a hydrogel, however, such focal adhesions are not promptly formed in the very first phase of the encapsulation. That is why cells appear rounded initially for a few days. After surviving in the hydrogel environment for a few days, cells start secreting extracellular matrix components along with partial local digestion of the collagen in the hydrogels. This is achieved by a combined secretion of MMPs in the extracellular matrix and attachment of the cells to the RGD signatures in GelMA and newly secreted ECM components. This creates a niche for cells in the erstwhile difficult environment. Different cells would mold the hydrogels differently according to their own specific milieu of secreted proteins and the rate of digestion of the hydrogels. As shown in Figures 4 and 5, cells appear small in the gels initially (Day 1, Figure 5, upper panel). Two different behaviors are apparent with the passage of time. First, the cells migrate out of the GelMA molds and patterns and gradually cover the free area on the surface of the culture plates. Second, the cells appear to change their shapes, which is due to the building of a more favorable environment for the cells to attach to the matrix.

In Figure 4, the differences between the hydrogels patterned on uncoated and PEG/GelMA-coated surfaces are very apparent. As previously stated, PEG/GelMA is intended to reduce the migration of the cells out of the hydrogel patterns. PEG/GelMA is more hydrophobic and does not allow ready attachment of the cells as compared to the uncoated cell culture-treated surfaces. Consequently, the extra spaces outside of the patterns in uncoated surfaces are filled with the RPE (green) and Müller (red) cells by Day 12 (Figure 4A–C, lower panels), whereas these spaces are relatively empty on PEG/GelMA-coated surfaces (upper panels). The 3D organizations of these cells in the hydrogel patterns show the cellular distribution and size differences clearly (Figures 4 and 5). Comparison of RPE and Müller cells shows that more Müller cells stay inside the hydrogels, whereas more RPE cells prefer to migrate outside to the 2D surfaces. This reflects the innate differences in the behavior of the two types of cells in the hydrogel. It appears Müller cells adapt better to the hydrogel environment in 3D and carve a niche for themselves, whereas RPE cells make their way out of the 3D hydrogels faster. The migration of RPE cells, however, is discouraged on PEG/GelMA-coated surfaces. 3D representations with angular rotations of the cultures are shown in videos 1–2. Video 1 shows cellular profiles and distributions within the hydrogels on Day 3 on uncoated (left panel) and PEG/GelMA-coated surfaces (right panel), whereas video 2 shows the cellular profiles and distributions on Day 12. The differential migration on Day 3 and Day 12 is apparent between the settings. It seems that Müller cells exhibit better retention than the RPE inside the hydrogels.

Inherently, RPE and Müller cells are structurally and functionally divergent. Müller cells mostly occupy the core of the retina, interacting with and supporting multiple cell types, whereas RPE cells constitute a single cell layer of pigmented epithelium. Our system recapitulated the innate tendencies of RPE and Müller cells in native tissue. RPE cells exhibited urgency to exit the hydrogel to reach the 2D surface where they interact with each other to form a single-layered epithelium-like structure, while Müller cells showed a higher retention within GelMA, a prelude to their interspersed organization within the the retina, supporting other components of the neural retina. Previously, it has been reported that when different cell populations are mixed and studied for relative differences in migration, there are marked differences in RPE and retinal glial cells.^21^ RPE and Müller cells exhibit different integrin combination expressions, which could be responsible for their differential attachment to the GelMA and migration rates out of the scaffold.^22,23^

### Relative Organization within Hydrogels

**Figure 6:**
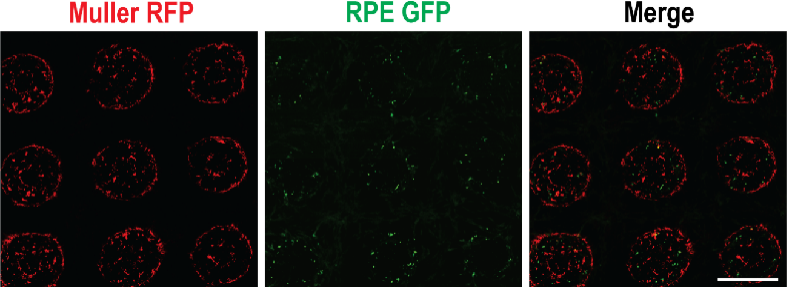
Relative positions of RPE1 cells (green) and Müller cells (red) on Day 12 of coculture.

Müller cells not only adhere better to the hydrogels but also organize themselves in a clear pattern by occupying the space along the edges of the patterns of hydrogels (Figure 6). More Müller cells appear on the edges, whereas the RPE cells that remain within the hydrogels appear to be randomly scattered (Figure 6). Whether RPE cells would occupy a niche within GelMA-based hydrogels is not clear with the current data, given the higher rate of outward migration by RPE cells. Outside of the hydrogels, RPE and Müller cells appear scattered and occupy the empty 2D surface.

We observed a higher accumulation of Müller cells on the edges than on the central parts of the hydrogel. Whether Müller cells acquire this orientation as an innate ability is not clear. A motility-dependent explanation, however, could be that it is due to the retardation of cellular migration when the cells arrive at the edges of the hydrogel. Reduced motility close to the edges will increase the possibility of encountering other less motile cells in the vicinity, thus increasing the number of cell–cell contacts. Since the cells tend to have an initial random migration, with slowing down at the edges over time, more and more cells get stuck at the peripheral edges of the hydrogel patterns.

### Interactions among the Two Types of Cells

**Figure 7:**
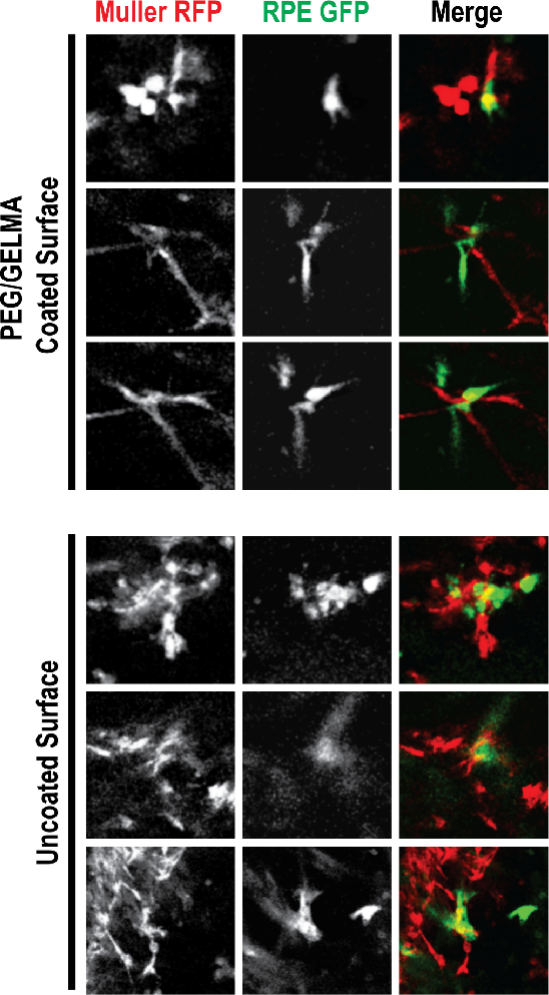
Examples of cellular proximity and interactions between RPE1 and Müller cells in the coculture. Upper panel: PEG/GelMA; lower panel: GelMA alone.

We intended to see if the RPE and Müller cells interact with each other in the hydrogel environment and to understand the nature of these interactions. As is clear from the earlier figures, RPE cells tend to migrate out of the hydrogels at a faster rate, providing less-than-optimal conditions for the two cell types to interact with each other. PEG/GelMA coating reduces the migration of RPE cells out of the hydrogels and may enhance the chances of interaction between the two types of cells. We observed random yet sparse interactions of the two types of cells in the hydrogels in both configurations (Figure 7). With the current data sets, we are limited in our ability to quantify the extent of interactions between the two cells. It is our understanding that reducing the extent of outward migration of RPE cells can enhance the interaction probabilities of the two cell types.

### RPE Spheroid Incorporation and Interaction with Müller Cells

We observed that the coculture of RPE1 and Müller cells in the GelMA-based hydrogels shows limited interaction between the two types of cells. This difference in the number of RPE1 and Müller cells inside the hydrogel could be due to several factors. Cells need to carve out a niche for themselves inside the hydrogel. This primarily involves an enrichment of the hydrogel with the secreted components of the extracellular matrix reminiscent of the native tissue, attachment of the cells to the extracellular matrix, and gradual digestion of the hydrogels by the cells. Proper attachment and close-to-native shape acquisition are required for the cells to proliferate and are usually mediated through integrin-dependent focal adhesion formation in the context of embryonic stem cells.^24^ Many aspects of the cell cycle are dependent on the dynamic attachments of cells to the matrix through integrins.^25^ In particular, cytokinesis and the G1 to S transition are dependent on the integrin attachment, with growing evidence for the G2 to M transition as well as the early mitotic phase.^25^ A recent preprint identified a new class of integrin-based adhesion, the curved adhesions that mediate attachment to the matrix in 3D.^26^ Curved adhesions primarily consist of α_V_β_5_ and are molecularly distinct from focal adhesions and clathrin lattices.^26^ Although GelMA consists of RGD signatures for integrin attachments, cells secrete their own specific milieu of ECM components that mediate their carving a niche within the 3D matrix. In a tissue environment, the ECM is made due to a concerted secretion from various types of cells, and this native ECM could be different from that made by the secretion of a limited variety of cells onto GelMA, as in the present setting.

Another difference between the two cells is their motility. Differential secretion of ECM and the dependence of the two types of cells on this secreted ECM can attribute differential motility and migration behavior to the two types of cells. It may also reflect the innate preference of the cells, pertaining to their anatomical and cellular localization. Clinical studies have shown that one of the stress responses exhibited by RPE cells during AMD is intraretinal migration^27,28^ and AMD-associated intraretinal RPE migration occurs at various stages of the disease progression.^29,30^ RPE migration into the neurosensory retina occurs prior to chorioretinal atrophy^31,32^ and eventually causes the death of RPE cells.^27^ Intraretinal hyperreflective foci (HRF) are reported in retinopathies, including AMD, retinitis pigmentosa, and diabetic neuropathy, among others, and these HRF are strongly believed to be a result of intraretinal RPE migration.^31,33,34^

Müller cells display remodeling within the retina during pathogenesis. Studies in rats and mice show that GFAP reactivity of Müller cells increases tremendously after injury.^35,36^ Geographic atrophy (GA) in the retina, where RPE and photoreceptor cells atrophy, shows locally activated Müller cells (positive for GFAP and Vimentin) extending beyond the ELM and forming gliotic membranes,^37^ most likely to replace the loss of the photoreceptors, which are their usual binding partners. In retinitis pigmentosa, there appear glial extensions termed “seals,” which are layers of Müller cell processes.^38−40^ The difference in the retention within the gel and motility of the two types of cells could be due to their innate roles and organization within the retina. To further elaborate, RPE cells may not be very adaptive to persist in a 3D hydrogel and would prefer to form a single cell layer on a 2D planar surface. It may also be that one type of cell could be more motile and less dependent on the secreted ECM than the other during migration. Differences in the number of cells remaining inside the GelMA hydrogel after 12 days of incubation could also be a result of differences in the proliferation of the two types of cells and their motility within and out of the hydrogels. We chose to test attributing this limitation to the difference in the rates of migration of the two types of cells out of the hydrogels. While both RPE1 and Müller cells exhibited outward movement of the cells, we observed a higher number of RPE cells outside of the hydrogel patterns onto the 2D surface, whereas many more Müller cells stayed inside the hydrogel (Figures 4 and 5). Due to this differential retention of the cell types within the hydrogel, the interaction between the two cell types was rather limited.

**Figure 8:**
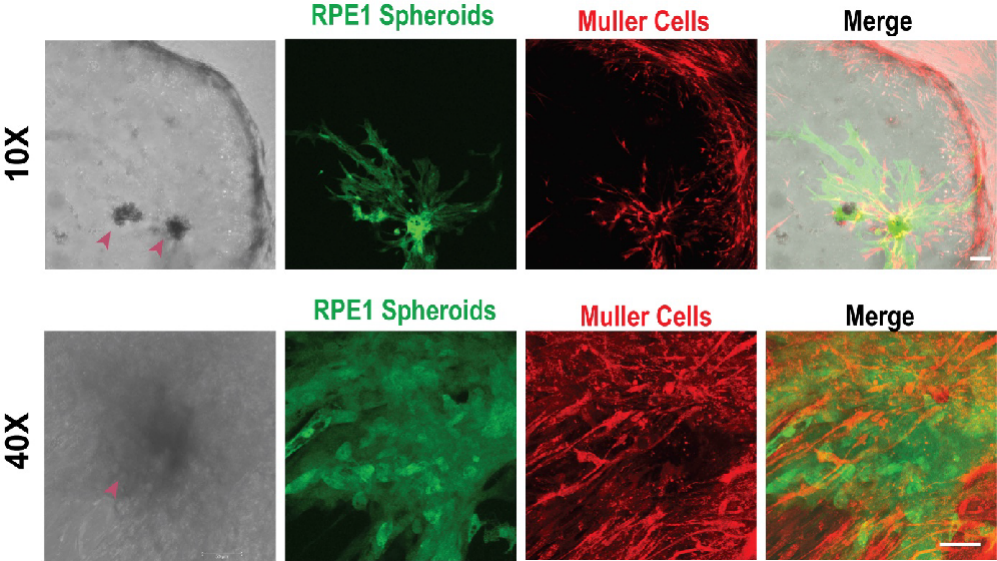
RPE1 spheroids (green) and free Müller cells (red) coculture. Interactions between RPE cells emerging from spheroids (magenta arrowheads) and Müller cells are shown here. The upper panel shows RPE cells interacting with Müller cells at 10× magnification. Scale bar: 100 μm. The lower panel shows the same at 40× magnification. Scale bar: 50 μm.

During disease and retinal injury, RPE cells exhibit intraretinal migration, and Müller cells undergo remodeling to grow beyond their canonical boundaries, thus increasing the possibility of interactions between these two cell types. We devised a different method to counter the differential migration rate and the resultant low interaction in the coculture to emulate remodeled intraretinal interactions after injury. We performed the spheroid generation of RPE cells, and these spheroids and free Müller cells were incorporated into GelMA with photomasks to generate hydrogel patterns. Spheroids were sparingly incorporated with only 0–2 spheroids per GelMA pattern. RPE1 spheroids displayed relatively stable sizes within the first week of incorporation in GelMA. This was congruent with our plan to suppress the migration of RPE1 cells out of the spheroids. By Day 27, we saw RPE1 cells exuding out of the spheroids and spreading within the 3D hydrogels (Figure 8 and Videos 3–5). At higher resolution (40×), the interaction between the RPE1 and Müller cells is clearly visible (Figure 8, lower panel). We usually see the bulk of Müller cells migrating toward the edges of the hydrogel patterns (Figure 6) and very few cells in the core of the hydrogel. However, the coculture of RPE1 spheroids and Müller cells indicates that many more Müller cells got trapped with the spreading RPE1 cells from the spheroids (Figure 8 and Videos 3–5). Thus, we were able to retard RPE1 migration from the hydrogels by incorporating them into spheroids, which led to the enhanced interaction between the RPE1 and Müller cells.

Certain retinal organoids derived from iPSCs have the advantage of emulating the native tissue more closely. They contain nearly all major types of cells in the native retina, including photoreceptors, amacrine cells, retinal ganglion cells, bipolar cells, horizontal cells, and Müller cells. The development of retinal organoids usually takes months with a painstaking series of chemical treatments.^41,42^ In comparison, our system is a hydrogel-based scaffold system that provides a minimal platform on which further complexity can be introduced with relative ease. Being transparent and accessible for imaging, gradual homotypic and heterotypic cellular interactions can be studied with relative ease. Biomechanical mimicry due to ECM-like interactions and the possibilities of further customizability and supplementation of GelMA add a whole new layer of advantages. Our multiplex system can be conveniently adapted to study patient-derived tissue clusters and primary cells due to the ease of incorporation within the platforms. Additionally, our system opens up possibilities for use in bioactive material and drug screenings due to the ease of chemical supplementation.

## Conclusion and Future Directions

Creating a reconstituted in vitro model of retinal composition is important to understand the intercellular interactions and disruptive dynamics of the tissue in disease. Keeping this goal in mind, we aimed at creating a coculture system of RPE and Müller cells in a 3D hydrogel-based multiplex platform, which imparts an innate replication of the experiments. Such a system can come handy while performing a high-throughput study, a CRISPR screening, or drug screening. We have shown that RPE and Müller cells are both capable of showing proliferation while encapsulated in GelMA. RPE1 and Müller cells displayed differential retention within GelMA over time, with RPE1 cells showing a greater outward migration, while Müller cells showed better retention within the hydrogel. We also showed that prestamping a PEG/GelMA mix to shield the cell culture surface discouraged cellular migration out of the hydrogels and enhanced their retention. RPE1 and Müller cells showed interactions within the hydrogel, albeit limited. Due to the differential retention and migration of the two types of cells, they did not have optimal possibilities to interact with each other. We devised an alternative method of incorporating RPE1 cells into spheroids before coculturing them with Müller cells. This decreased the exodus of RPE cells from the hydrogel and gave a better chance for Müller cells to interact with the RPE1 cells, slowly exiting the spheroids.

Thus, our study provides a useful method of coculturing retinal components in a 3D hydrogel-based multiplex platform. This system can also be easily utilized to incorporate patient cell-derived spheroids and organoids within the hydrogel to observe the organizational behavior of cells.

## Supporting Information Available

3D organization of RPE (green) and Müller (red) cells in multiplex hydrogels on Day 3 after encapsulation. Rotation angle 360° highlighting the overall distribution of the two cell types in coculture. Left panel: coculture on the uncoated surface. Right panel: coculture on the PEG/GelMA-coated surfaces. Scale bars: 1 mm (AVI)
3D organization of RPE (green) and Müller (red) cells in multiplex hydrogels on Day 12 after encapsulation. Rotation angle 360° highlighting the overall distribution of the two cell types in coculture. Left panel: coculture on the uncoated surface. Right panel: coculture on the PEG/GelMA coated surfaces. Scale bars: 1 mm (AVI)
Interaction of RPE-derived spheroids and Müller cells in the coculture. RPE cells migrating out of the spheroids and interacting with Müller cells on the 3D hydrogel. Image taken 27 days after RPE spheroid and Müller cell initial incorporation in hydrogels with 10× objective (MP4)
40× objective (MP4)
A close-up depiction of interactions between RPE spheroids and Müller cells at 40× (MP4)

## Supplementary Material

mt4c01376_si_001.avi

mt4c01376_si_002.avi
